# Understanding salinity stress responses in sorghum: exploring genotype variability and salt tolerance mechanisms

**DOI:** 10.3389/fpls.2023.1296286

**Published:** 2024-01-09

**Authors:** Ahmad Rajabi Dehnavi, Morteza Zahedi, Agnieszka Piernik

**Affiliations:** ^1^ Department of Geobotany and Landscape Planning, Faculty of Biology and Veterinary Sciences, Nicolaus Copernicus University in Torun, Torun, Poland; ^2^ Department of Agronomy and Plant Breeding, College of Agriculture, Isfahan University of Technology, Isfahan, Iran

**Keywords:** antioxidant enzymes, osmotic adjustment, photosynthetic pigments, salinity, sorghum genotypes

## Abstract

Salinity, a significant abiotic stressor, adversely affects global plant growth. To address this, monitoring genetic diversity within a plant species germplasm for salt tolerance traits is vital. This study investigates the responses of ten sorghum genotypes to varying salt stress levels (control, 60 mM NaCl, and 120 mM NaCl), aiming to assess genetic diversity. Using a randomized complete block design with three replications and a split-plot arrangement, salt treatments were assigned to main plots, and genotypes were placed in sub-plots. Physiological attributes, including photosynthetic rate, stomatal conductance, CO_2_ concentration, leaf area index, chlorophyll concentrations, and antioxidant enzyme activity, were measured during the 50% flowering stage. Fresh forage yield was evaluated at the early dough stage, while dry forage yield and sodium/potassium concentrations were determined post-drying. Salinity induced 10–23% and 21–47% reductions in forage fresh yield at 60 mM and 120 mM NaCl, respectively, across sorghum genotypes. Forage dry yield also declined by 11–33% at 60 mM NaCl and 30–58% at 120 mM NaCl. Increased oxidative stress markers, proline, soluble carbohydrates, and antioxidant enzyme activity accompanied salinity. Genotypes exhibited diverse responses, with Payam showing significant chlorophyll and yield reductions at 60 mM NaCl and notable stress indicators at 120 mM NaCl. Pegah and GS4 demonstrated robust osmoregulation. In stress tolerance indices, Sepideh excelled at 60 mM NaCl, while GS4 outperformed at 120 mM NaCl. Pegah demonstrated high tolerance at 120 mM NaCl. Our findings highlight the importance of combating oxidative stress, managing water-related stress, and maintaining ionic homeostasis for sorghum’s salt stress resilience. Key indicators like K/Na ratio, MDA, MSI, SOD, and proline effectively differentiate between tolerant and sensitive genotypes, offering valuable insights for sorghum breeding. Salt-tolerant sorghum genotypes exhibit stable photosynthesis, improved stomatal function, and membrane integrity through efficient osmotic regulation and robust antioxidant enzyme activity. This capability enables them to sustain performance, minimizing final product loss. The results suggest cultivating salt-tolerant sorghum in saline areas for increased sustainable production, with Pegah and GS4 emerging as promising candidates for further testing in salt-affected environments to obtain reliable yield data.

## 1 Introduction

The global agricultural landscape is grappling with a pressing issue known as salinity, which characterized by the accumulation of soluble salts in soil and irrigation water. This condition poses a significant challenge with profound implications for food security (Fadl et al., 2023). Approximately 20% of cultivated lands worldwide are affected by salinity, and this problem is particularly acute in arid and semi-arid regions, characterized by high evaporation rates and suboptimal irrigation practices (Mukhopadhyay et al., 2021). Coastal areas, contending with saltwater intrusion, face an added layer of vulnerability (Epanchin-Niell et al., 2023). The escalation of salinity levels over the past decade is the result of a complex interplay of factors, including climate change, excessive water usage, deforestation, industrial activities, and pollution, which highly affected food security and plant production worldwide (Phour and Sindhu, 2023). Salinity adversely impacts crops through osmotic stress, ion toxicity, mineral deficiencies, and various physiological and biochemical impairments (Balasubramaniam et al., 2023). Furthermore, the excessive salt content triggers the generation of reactive oxygen species (ROS) within plant cells. This oxidative stress leads to membrane damage and exacerbates the detrimental impact of salinity on crops (Hasanuzzaman et al., 2021). Additionally, it disrupts soil structure, impeding root penetration, nutrient uptake, and microbial activity, thereby negatively affecting the overall soil ecosystem (Naorem et al., 2023). Ultimately, these multifaceted effects of salinity stress culminate in reduced crop yields in terms of quality and quantity, underscoring the pressing need for strategies to mitigate the adverse consequences of salinity on agriculture.

Monitoring genetic diversity within a plant species germplasm for salt tolerance traits stands out as a crucial approach to mitigating salt stress (Amombo et al., 2022; Kaur et al., 2023). This method helps pinpoint genotypes that exhibit greater sustainability and superior performance compared to their counterparts (Ashraf et al., 2006). It also serves as a foundational strategy for selecting and developing salt-tolerant varieties that can thrive in saline environments, ultimately contributing to improved crop yields and agricultural sustainability. Numerous studies have consistently documented substantial genotypic variation in salt tolerance across various plant species (Manzoor et al., 2023; Sagar et al., 2023; Shams and Khadivi, 2023). It well documented that salt-tolerant plants employ a diverse array of defense mechanisms in response to salinity stress to ensure their survival and promote growth. One critical aspect of this defense strategy is the maintenance of cellular ion balance, particularly the equilibrium between sodium (Na^+^) and potassium (K^+^), which is essential for the plant viability in saline conditions (Wakeel et al., 2011; Pantha et al., 2023). In addition, plants have developed intricate defense mechanisms to counteract the detrimental impacts of ROS induced by environmental stress, including salinity (Sachdev et al., 2021). This defense system relies on both non-enzymatic antioxidants and antioxidant enzymes working in tandem to neutralize and eliminate ROS accumulation triggered by salt stress (Lamalakshmi Devi et al., 2017). Furthermore, plants accumulate compatible solutes, such as carbohydrates, amino acids, proline, and proteins, as part of their defense strategy (Garcia-Caparros et al., 2021). These solutes play a crucial role in osmotic regulation, ensuring proper water uptake and cell turgor (Sanders and Arndt, 2012). Ultimately this multifaceted defense system enables plants to adapt and thrive in challenging, saline environments.

Sorghum (*Sorghum bicolor* L.) is the fifth most important cereal crop in the world, which plays an important role in feeding the growing world population (Iqbal, 2015). Sorghum is known for its adaptability to various stressors, including salinity. Generally, sorghum is moderately salt-tolerant and considered to tolerate salinity levels up to 70 mM NaCl (Allen et al., 1998). However, the response to salinity stress can vary among different sorghum genotypes.(Shakeri and Emam, 2018; Rajabi Dehnavi et al., 2020). Investigating these genotype-specific responses is vital for sustainable agriculture and food security by enhancing sustainable sorghum production in salt-affected areas (Hossain et al., 2022). In addition, it can aids in understanding the genetic basis of salt tolerance in sorghum and contributes to breeding programs by uncovering genetic traits and markers associated with salt tolerance (Afzal et al., 2023). However, there is limited knowledge regarding the mechanisms involved in salt-tolerant genotypes.

Thus, building upon our prior research on genetic diversity within sorghum plants germplasm for salt tolerance at germination and seedling stages (Rajabi Dehnavi et al., 2020), this study aims to investigate sorghum genotypes under field conditions. The focus is on forage yield under salt stress and the mechanisms involved in salt tolerance. The experimental objectives are to explore how (1) salinity stress impacts the physiological attributes and forage yield of sorghum genotypes, (2) different sorghum genotypes demonstrate variations in their tolerance to salinity, and (3) salt-tolerant sorghum genotypes exhibit efficient defense mechanisms against salt stress.

## 2 Materials and methods

### 2.1 Experimental conditions

The research was conducted in the summer of 2021 at the research farm of Isfahan University of Technology, located in the Larek region of Najaf Abad City, Iran. The study area has a semi-arid climate with dry summers and an elevation of 1630 meters above sea level. The average annual temperature is 15.2°C, with the hottest months being July and August and the coldest months being December, January, and February. The highest recorded temperature was 42.5°C, while the lowest was -18.5°C. The average annual rainfall is 150.9 mm, and during the sorghum growing season, the average maximum and minimum temperatures were 34.2°C and 19.5°C, respectively (**Supplementary Figure 1**).

The soil texture of the farm was classified as loam-clay, with an apparent specific gravity of 1.3 g cm^-3^ and pH of 7.5. Before conducting the experimental treatments, the soil characteristics were assessed at 0 to 50 cm depth in the field experiment (**Table 1**).

**Table 1:** Characteristics of the soil used in the field experiment at a depth of 0 to 50 cm.

EC (dS/m)	Total N (%)	K (mg/kg)	P (mg/kg)	Mg (mg/kg)	Fe (mg/kg)	PWP (%)	FC (%)
2.1	0.1	150	19.1	48	8.5	10	23

### 2.2 Experimental design

This experiment used a split-plot design within a randomized complete block design with three replications. The main factors included three irrigation water salinity levels (control, 60 mM NaCl, and 120 mM NaCl). The sub-factors consisted of ten genotypes (Pegah, Speed feed, Jumbo, Kimia, Sepideh, and Payam, GS4, KGS29, KGS23, and MGS5) obtained from the Seed and Plant Improvement Institute in Karaj, Iran. These sorghum genotypes show great promise in enhancing sustainable sorghum production in arid and saline regions like Iran. Exhibiting high yields, functional stability, and adaptability to challenging environmental stress conditions, these genotypes have become commercially cultivated by sorghum growers in the region. Their successful adoption underscores their recognized value and practical utility in addressing the specific agricultural challenges posed by aridity and salinity in Iran. Seeds were sown during the last week of June 2021. Each main plot measured 30m × 2.5m, with sub-plots of four rows of crops spaced 75 cm apart.

### 2.3 Irrigation and salinity application

In this investigation, the irrigation system is configured in a drip tape format, utilizing a flow meter to regulate both the volume and frequency of irrigation. Initially, during the first two weeks after planting, the plants in the field received consistent and uniform irrigation, maintaining soil moisture at 40% of the total available water (TAW) discharge in the non-saline control treatment. Following the complete establishment of the plants, saline irrigation water treatments were introduced and persisted until mid-November 2021. Throughout the entire growth phase of the plants, the irrigation strategy adhered to the soil’s moisture curve, tailored to its texture, with the objective of achieving a 55% TAW discharge in the non-saline control treatment.

To determine the required water amount for each plot, TAW in the field was calculated in mm using Equation 1 (Allen et al., 1998).


(1) $$TAW\;=\;\left(\theta_{FC}-\;\theta_{PWP}\right)\times 10\rho_{b}\times D_{rz}$$

${\text{θ}}_{\text{FC}}=$ moisture percentage of soil water weight in the field capacity

${\text{θ}}_{\text{PWP}}=$ Moisture percentage of soil water weight at permanent wilting point

${\text{ρ}}_{\text{b}=}$ mass of soil volume(g/m^3^)

${\text{D}}_{\text{rz}}=\;$ depth Development plant (cm)

The readily available water (RAW), representing the fraction of TAW easily absorbed by the plant without stress from the root development zone, was calculated using Equation 2 (Allen et al., 1998).


(2)   RAW=Total available water × ρ

Here, ρ for the sorghum plant was considered as 55% of TAW discharge (Allen et al., 1998). The irrigation level was strategized according to the percentage of maximum allowable depletion, as outlined by Allen et al. (Allen et al., 1998). This approach guaranteed that irrigation took place following the discharge of 55% of TAW in the non-saline control treatment.

To establish the irrigation timing, the humidity and corresponding suction needed to delineate the soil moisture curve were computed using a pressure plate device. The alteration in soil moisture was tracked using a tensiometer, initiating two days post-irrigation and persisting until the subsequent irrigation event.

The determination of the water volume needed for each irrigation level (TVW), aimed at augmenting water content within the root development area (0.5 m), was accomplished using Equation 3 (Allen et al., 1998).


(3) $$\;\text{TVW}=\;\;\frac{RAW\times Plot\;area}{Irrigation\;efficiency\;}\;$$

In this context, TAW is measured in cubic meters (m³), the plot area is in square meters (m²), and f represents the percentage of moisture discharge from the Total Available Water (TAW) set at 55 percent, delineating the irrigation levels in this experiment. Assuming an irrigation efficiency of 70% during the growing season (Allen et al., 1998), the volume of irrigation water for each plot is calculated. Subsequently, the amount of salt required to achieve the desired salinity levels (60 and 120 mM NaCl) is computed in kilograms per liter (kg/l). The precise salinity treatment is executed through the strip irrigation system connected to the reservoir. To avert osmotic shock to plants, the salt for the salinity treatment is gradually introduced into the plant growth medium in three stages. To prevent the accumulation of salt and maintain soil salinity at an approximately constant level, we adhered to the salinity regulation approach outlined in the Food and Agriculture Organization (FAO) guideline (Ayers and Westcot, 1985). This method focuses on leaching salts out of the root zone before they reach the target soil electrical conductivity (EC). In line with this strategy, we calculated the percentage of drainage as the ratio of total irrigation water, taking into consideration the soil textire, the initial EC of the soil, the volume and EC of the irrigation water, and the targeted final EC of the soil. Prior to each irrigation cycle, we conducted a comprehensive assessment of soil salinity up to the depth of root development (50 cm). The measured soil EC from each evaluation served as the initial soil EC for that particular irrigation round.Throughout the salt treatment process, the EC for the 60 mM NaCl treatment and post-experiment was approximately 6.84 dS/m, while for the 120 mM NaCl treatment, it reached around 12.4 dS/m. These values reflect our commitment to controlling and maintaining specific salinity levels in the soil throughout the experiment.

### 2.4 Traits measured at the 50% flowering stage

A destructive sampling method was employed during the 50% flowering phenological stage to assess the desired traits. Sampling took place from late August to mid-September 2021, with the timing customized for the specific genotypes under investigation.

#### 2.4.1 The relative water content

The flag leaf was harvested in the morning and packed in a nylon bag for preservation. Healthy leaf pieces were selected, weighed, and placed in Petri dishes with distilled water for 4 hours at 23°C. After removing excess moisture, the leaves were weighed again to determine accumulated weight. Next, the leaves were dried at 70°C for 48 hours, and their dry weight was measured. RWC was calculated using Equation 4 (Smart and Bingham, 1974) based on these measurements.


(4) $$\;RWC=\left(\frac{leaf\;fresh\;weight\;-leaf\;dry\;weight}{turgid\;leaf\;weight-leaf\;dry\;weight}\;\right)\times \;100\;$$

#### 2.4.2 Concentration of hydrogen peroxide

H_2_O_2_ concentration was determined following the Velikova et al. method (Velikova et al., 2000). Plant parts were treated with 0.1% trichloroacetic acid, centrifuged at 4000 rpm for 15 minutes (4°C), and mixed with zinc solution, potassium phosphate buffer, and potassium iodide. Absorbance at 390 nm was measured using a spectrophotometer. H_2_O_2_ concentration was calculated with an extinction coefficient of 0.28 mM^-1^ cm^-1^ and expressed as µmol/g FW.

#### 2.4.3 Malondialdehyde content

To evaluate lipid peroxidation in cell membranes induced by salinity in sorghum plants, the concentration of MDA was quantified following the Davey et al. method (Davey et al., 2005). Plant extract (0.1** g**) was homogenized with 0.5 ml of 0.1% TCA, centrifuged, and mixed with 20% Trichloroacetic Acid (TCA) and 0.5% Thiobarbituric Acid (TBA). After heating and cooling, the MDA-TBA complex was measured at 532 nm. The MDA content was calculated using an extinction coefficient of 155 mM^-1^cm^-1^ and expressed as nM MDA/g FW.

#### 2.4.4 Membrane stability index

Fresh leaves (0.1g) were immersed in 10 ml of double distilled water for the analysis. After 30 minutes at 40°C, the electrical conductivity (EC) was measured using an EC meter (C1). Then, the sample was exposed to 100°C for 15 minutes, and the electrical conductivity was measured again (C2). With these values, the MSI was calculated and expressed as % using Equation 5 (Saqib et al., 2011).


(5) $$\text{MSI}=\left(1-\frac{C1}{C2}\;\right)\times \;100\;$$

#### 2.4.5 Photosynthetic attributes

The photosynthetic rate (Pn), stomatal conductance (Gs), and intercellular CO_2_ concentration (Ci) were measured between 9 a.m. and 11 a.m. using a portable photosynthetic system gas analyzer (LI-COR 6400, LI-COR, Lincoln, NE, USA) and expressed as µmol/(m²·s).

#### 2.4.6 Leaf area index

A destructive method was employed to determine the leaf area (LA), and an electronic leaf area meter (model Winarea-ut-11, made in Iran) was used. The LA was measured in cm^2^/plant. Subsequently, the LAI was calculated, representing the LA (on one side only) relative to the land area occupied by the crop.

#### 2.4.7 Photosynthetic pigments content

Chlorophyll concentration was determined using Lichtenthaler and Buschmann method (Lichtenthaler and Buschmann, 2001). Leaves (0.5 g) were crushed with 10 mL of 80% acetone. The mixture was filtered using Whatman paper until the residue became white. The extract was centrifuged at 5000 rpm for 15 minutes. Each test tube was adjusted to a 10 ml volume with 80% acetone. Absorption was measured at 663 nm, 646 nm, and 470 nm (Lichtenthaler and Buschmann, 2001) wavelengths using a spectrophotometer. Equation 6, 7, and 8 were used to calculate chlorophyll a, b, total and leaf carotenoid concentrations in mg/gFW


(6) $$\text{Cholorophyll}\left(a\right)=\frac{\left[\left(12.21\times \text{Abs}663\right)-\left(2.81\times \text{Abs}646\right)\times \text{mlAseton}\right]}{\text{mgLeaf}}$$

(7) $$\text{Cholorophyll}\left(\text{b}\right)=\frac{\left[\left(20.13\times \text{Abs}646\right)-\left(5.03\times Abs663\right)\times \text{mlAseton}\right]}{\text{mgLeaf}}$$

(8) $$\text{Carotenoids}=\frac{\left(\left[\frac{1000\times \text{Abs}470-3.27\left(\text{Chla}\right)-104\left(\text{Chlb}\right)}{227}\right]\times \text{mlAseton}\right)}{\text{mgLeaf}}$$

In these Equations, 646 Abs, 663 Abs, and 470 Abs absorb at 646, 663, and 470 nm wavelengths, respectively.

#### 2.4.8 Proline content

To determine the proline content (P), the method of Bates et al. was followed (Bates et al., 1973). Plant tissue (0.5g) was ground with 10ml 3% sulfosalicylic acid, and the extract was centrifuged. Two milliliters of the filtered extract were mixed with ninhydrin reagent and glacial acetic acid. After heating and cooling, toluene was added, and the red-colored upper phase containing P was separated. P standards were prepared, and the absorbance was measured at 520 nm using a spectrophotometer, with toluene as the blank.

#### 2.4.9 The content of soluble carbohydrates

To determine the soluble carbohydrate content (Carbo), the method described by Irigoyen et al. was followed (Irigoyen et al., 1992). Leaf tissue (0.5g) was pounded with 5 ml of 95% ethanol to obtain an alcoholic extract. The upper phase was separated, and sediments were washed with 70% ethanol. The combined upper phase was centrifuged, and a portion of the supernatant was transferred to a test tube. Fresh anthrone solution was added, and after heating and cooling, the absorbance was measured at 625 nm using a spectrophotometer. Glucose standard solutions were prepared for a standard curve.

#### 2.4.10 The activity of antioxidant enzymes

To determine the specific activity of antioxidant enzymes, 100 mg of plant tissue was homogenized with 1 ml of extraction buffer (1% polyvinylpyrrolidone and 0.5% Triton X100 in 100 mM potassium phosphate buffer, pH = 7). The transparent supernatant was collected for enzyme activity measurement after centrifugation at 15,000 rpm and 4°C for 20 minutes.

##### 2.4.10.1 The specific activity of catalase enzyme

The specific activity of the enzyme was measured using a modified method based on Alici and Arabaci (Alici and Arabaci, 2016). The assay involved mixing reaction buffer with enzyme extract and monitoring the change in absorbance at 240 nm over two minutes. The volumetric activity of the enzyme was calculated by dividing the enzyme activity by the reaction mixture’s volume. The specific activity of the enzyme was determined by dividing the volumetric activity by the protein concentration, measured using the Bradford method (Bradford, 1976).

##### 2.4.10.2 The specific activity of ascorbate peroxidase enzyme

APX activity was determined based on the Nakano and Asada method (Nakano and Asada, 1981), measuring the absorbance decrease at 290 nm. The reaction mixture comprised reaction buffer enzyme extract, and the change in absorbance at 290 nm was monitored over two minutes. The specific activity of APX can be calculated similarly to the CAT enzyme assay, dividing the volumetric activity by the protein concentration in the extract.

##### 2.4.10.3 The specific activity of superoxide dismutase enzyme

SOD enzyme activity was determined using a modified method by Giannopolitis and Ries (Giannopolitis and Ries, 1977). The activity was measured by inhibiting nitro-blue tetrazolium photoreduction at 560 nm. A reaction solution containing enzyme extract and various components was exposed to light for 15 minutes, and the absorption at 560 nm was measured. The specific activity of SOD can be calculated using the same method as the CAT enzyme assay, dividing the volumetric activity by the protein concentration in the extract.

### 2.5 Measured traits in the pulping stage of the seeds

#### 2.5.1 Quantitative characteristics of sorghum

To measure the total fodder yield, in the middle of November 2021, the fresh and dry yields were determined by harvesting the middle two rows of each plot, excluding two bushes at the beginning and end. Yields were reported as t/ha to indicate productivity.

#### 2.5.2 Concentration of Na^+^ and K^+^

Samples were dried, ground, and subjected to high-temperature treatment to convert organic material into ashes. The resulting ashes were dissolved in hydrochloric acid, filtered, and adjusted to a final volume. Concentrations of Na^+^ and K^+^ were measured using a Flame Photometer. A calibration curve was created using standard solutions to correct the data, and the Na^+^ and K^+^ concentrations were reported as mg/g DW.

### 2.6 Stress sensitivity index

The SSI of sorghum plants was calculated using Equation 9 (Fischer and Maurer, 1978). This indice provide quantitative measures of salt stress tolerance. A smaller SSI value indicates higher tolerance (Fischer and Maurer, 1978).


(9) $$SSI\;=\left(\frac{1-\frac{Ys}{Yp}}{1-\frac{\bar{Y}s}{\bar{Y}p}}\;\right)\;\;$$

In the equation, Ys represents genotype performance in a stressful environment, Yp represents genotype performance in a stress-free environment, $\bar{Y}$ s is the average performance of all genotypes in a stressful environment, and $\bar{Y}$ p is the average performance of all genotypes in a stress-free environment.

### 2.7 Salinity tolerance index

The salinity tolerance index of sorghum plants was calculated using Equation 10 (Fernandez, 1992). This indice offer quantitative assessments of salt stress tolerance, with a higher STI value indicating a higher level of overall tolerance (Fernandez, 1992).


(10) $$STI\;=\frac{Yp\;\times \;Ys}{{\left(\bar{Y}p\right)}^{2}}\;$$

Ys represents genotype performance in a stressful environment, Yp represents genotype performance in a stress-free environment, $\bar{Y}$ s is the average performance of all genotypes in a stressful environment, and $\bar{Y}$ p is the average performance of all genotypes in a stress-free environment.

### 2.8 Statistical analysis

Statistical analysis was performed using SAS version 9.4 (SAS Institute Inc., Cary, NC, USA) with two-way ANOVA to assess treatment variances and determine significance (p ≤ 0.05). *Post-hoc* analysis was conducted using the HSD test. Principal Components Analysis (PCA) was employed to understand the effects and generate a PCA diagram, utilizing Past software version 4.13. Hierarchical agglomerative cluster analysis was employed to illustrate genotype similarity, utilizing percent similarity as the measure of similarity and the unweighted pair group method for constructing the classification tree. Cluster analysis was performed using Past software version 4.13.

## 3 Results

### 3.1 Water relations

**Figure 1:**
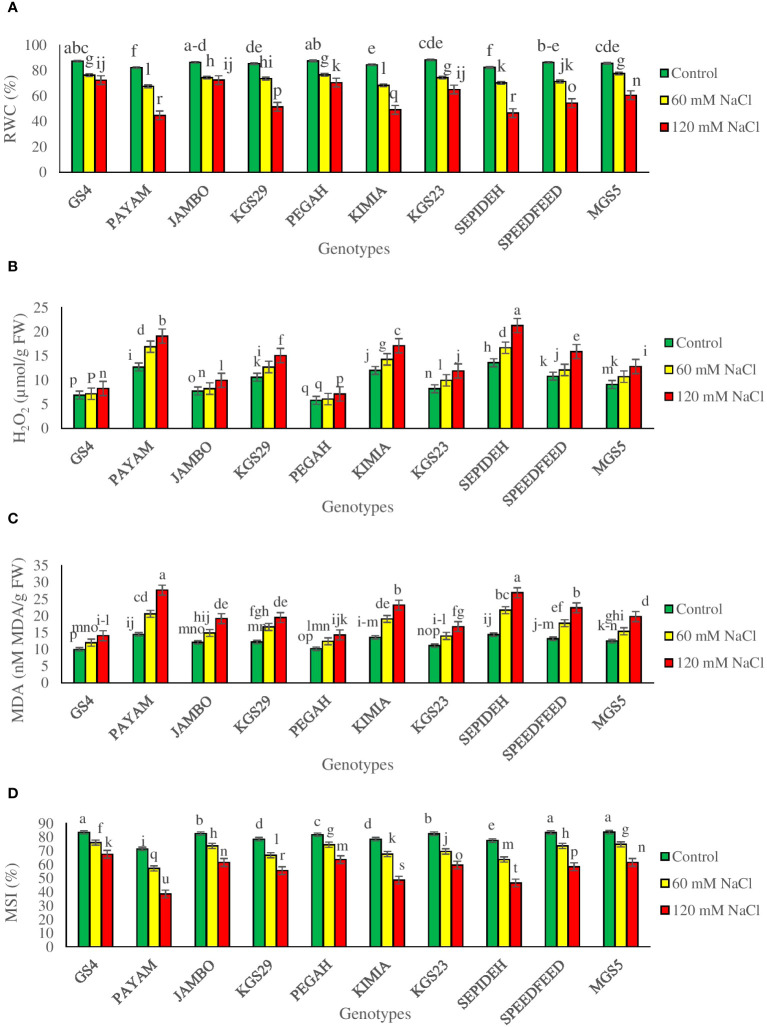
**(A)** relative water content (RWC), **(B)** hydrogen peroxide (H_2_O_2_), **(C)** malondialdehyde (MDA), and **(D)** membrane stability index (MSI) of sorghum genotypes for interaction of genotypes × salinity (0, 60 and 120 mM NaCl) at fifty percent flowering stage. Different letters indicate significant differences by HSD at p<0.05.

Evaluating RWC is crucial in understanding water dynamics and adaptive responses to salinity stress. The study demonstrated significant main effects of both salinity and sorghum genotypes, as well as notable interaction effects, particularly concerning Relative Water Content (RWC) (**Supplementary Table 2**). Salinity exerted a considerable impact on RWC across all sorghum genotypes, leading to a general reduction. Specifically, exposure to 60 mM NaCl resulted in a 14.6% decline in RWC, while 120 mM NaCl induced a more substantial reduction of 31.4% (**Table 2**). Furthermore, the main effects of genotypes revealed distinct variations in RWC values. Among all sorghum genotypes studied, GS4 and Pegah exhibited the highest RWC values, suggesting a comparatively better ability to maintain water content under salinity stress. In contrast, genotypes Payam and Sepideh displayed the lowest RWC values, indicating a potentially lower capacity to withstand the deleterious effects of salinity on water retention (**Table 2**). Furthermore, the interaction effects between salinity and genotypes reveal notable variations among the sorghum genotypes. Specifically, at 60 mM NaCl, Kimia demonstrates the most significant decrease in Relative Water Content (RWC) at 19%, while MGS5 exhibits a more moderate reduction at 9%. Moving to higher salinity levels, at 120 mM NaCl, Payam experiences the most substantial reduction in RWC at 46%, while Jumbo shows a comparatively lesser decrease at 16% (**Figure 1A**). This decline is attributed to osmotic stress and reduced water availability induced by elevated salinity. The distinct responses underscore the critical role of RWC as a discriminating parameter, shedding light on the unique adaptive mechanisms employed by each genotype to address challenges posed by salinity.

**Table 2:** Mean comparisons for different parameters of ten Sorghum Genotypes under Three levels of Salinity.

Trait	Salt stress (mM NaCl)			Sorghum Genotypes									
	Control	60	120	GS4	Jumbo	Pegah	KGS23	MGS5	Speed feed	Sepideh	Kimia	KGS29	Payam
RWC (%)	85.6 a	73.1 b	58.7 c	78.7 a	77.8 a	78.2 a	75.9 b	74.5 c	70.7 d	66.45 e	67.3 e	70.1 d	64.9 f
Na (mg/g DW)	0.278 c	0.367 b	0.694 a	0.346 f	0.350 f	0.320 g	0.417 e	0.416 e	0.453 d	0.546 b	0.455 d	0.521 c	0.637 a
K (mg/g DW)	8.72 a	6.55 b	4.89 c	7.76 b	7.58 c	8.27 a	7.41 d	5.97 g	6.62 f	5.33 h	6.92 e	5.89 g	5.47 h
K/Na	32.0 a	18.1 b	8.47 c	25.3 ab	24.2 b	27.4 a	20.1 c	16.7 de	18.4 cd	17.8 ef	17.8 d	16.4 de	14.0 f
H_2_O_2_ (µmol/g FW)	9.75 c	11.4 b	13.9 a	7.42 h	8.64 g	6.33 i	10.0 f	10.8 e	12.9 d	17.2 a	14.7 c	12.7 d	16.2 b
MDA (nM MDA/g FW)	12.3 c	16.5 b	20.4 a	12.0 e	15.4 c	12.2 e	13.9 d	15.9 c	17.8 b	21.0 a	18.7 b	16.0 c	20.9 a
MSI (%)	80.2 a	69.6 b	56.0 c	75.5 a	72.5 c	73.1 b	70.5 e	73.1 b	71.8 d	62.5 h	64.8 g	66.9 f	55.6 i
Pn (µmol m^–2^ s^–1^)	22.1 a	18.3 b	14.0 c	22.5 a	20.3 c	21.8 b	18.7 d	16.6 e	18.7 d	13.8 h	18.8 d	15.5 f	15.0 g
Gs (µmol m^–2^ s^–1^)	131 a	83 b	57 c	102 a	97 b	101 a	85 d	96 b	89 c	84 e	85 de	89 c	76 f
Ci (µmol m^–2^ s^–1^)	70 a	51 b	37 c	57 a	61 a	58 a	56 ab	60 a	43 d	50 bc	45 cd	50 bc	47 cd
LAI	6.49 a	5.77 b	5.06 c	6.90 a	6.61 ab	6.42 b	6.10 c	5.13 f	5.77 d	5.15 f	5.49 de	5.35 ef	4.82 g
Chl a (mg/g FW))	1.78 a	1.36 b	0.99 c	1.58 b	1.48 c	1.66 a	1.43 d	1.43 d	1.37 e	1.06 h	1.27 f	1.29 f	1.18 g
Chl b (mg/g FW)	0.532 a	0.384 b	0.265 c	0.545 a	0.496 b	0.524 a	0.377 e	0.416 d	0.458 c	0.260 h	0.291 g	0.333 f	0.239 h
Chl T (mg/g FW)	2.27 a	1.75 b	1.25 c	2.13 a	1.98 b	2.18 a	1.80 c	1.84 c	1.83 c	1.32 f	1.44 e	1.62 d	1.42 ef
Car (mg/g FW)	0.323 a	0.262 b	0.211 c	0.366 a	0.320 c	0.330 b	0.284 d	0.271 e	0.244 f	0.203 i	0.221 h	0.236 g	0.177 j
P (µmol/g FW)	10.6 c	15.9 b	19.6 a	24.8 a	17.3 c	24.8 a	19.5 b	13.1 e	14.5 d	7.51 i	11.8 f	10.9 g	9.57 h
Carbo (µmol/g FW)	6.72 c	7.84 b	9.76 a	8.06 d	7.69 e	7.11 g	11.0 a	8.57 c	6.41 h	9.49 b	7.72 e	7.27 f	7.71 e
CAT (U/mg protein)	0.398 c	0.695 b	0.778 a	1.02 b	1.00 c	1.12 a	0.707 d	0.566 e	0.495 f	0.296 i	0.321 h	0.422 g	0.280 j
APX (U/mg protein)	1.44 c	2.32 a	1.71 b	2.83 a	2.66 b	2.66 b	2.17 c	1.73 e	2.02 d	0.923 h	1.14 g	1.35 f	0.748 i
SOD (U/mg protein)	2.49 c	3.89 b	4.80 a	5.62 a	5.38 b	5.43 b	4.64 c	4.24 d	2.88 e	2.05 g	2.33 f	2.78 e	1.94 g
FFY (t/ha)	81.1 a	69.3 b	56.1 c	92.5 a	82.6 c	84.8 b	75.2 d	71.1 e	65.9 f	45.3 j	61.0 h	61.8 g	48.1 i
DFY (t/ha)	27.6 a	22.7 b	15.6 c	27.5 a	25.4 b	26.9 a	24.0 c	21.1 d	24.7 bc	13.0 g	19.8 e	21.2 d	16.2 f

RWC, relative leaf water content; Na, sodium content; K, potassium content; K/Na, K to Na ratios in shoot; H_2_O_2_, hydrogen peroxide concentration; MDA, malondialdehyde concentration; MSI, membrane stability index; Pn, photosynthetic rate; Gs, stomatal conductance; Ci, intercellular CO_2_ concentration; LAI, leaf area index; Chl a, chlorophyll a; Chl b, chlorophyll b; Chl T, total chlorophyll; Car, carotenoids; P, proline; Carbo, soluble carbohydrates; CAT, catalase; APX, ascorbate peroxidise; SOD, superoxide dismutase; FFY, fresh fodder yield; and DFY, day fodder yield. Different letters indicate significant differences by HSD at p<0.05.

### 3.2 ROS stress indicators

Our study investigated the impact of salt-induced reactive oxygen species (ROS) on cellular membranes, specifically hydrogen peroxide, along with examining membrane integrity (MSI) and damage levels (MDA). Significant main effects of salinity and genotypes, coupled with noteworthy interaction effects, particularly in relation to hydrogen peroxide, MDA, and MSI, were observed (**Supplementary Table 2**). Overall, salinity induced a reduction in MSI while elevating H_2_O_2_ and MDA levels across all sorghum genotypes. Specifically, exposure to 60 mM NaCl resulted in a 13.2% decrease in MSI and an increase of 16.9% in H_2_O_2_ and 34.1% in MDA (**Table 2**). At 120 mM NaCl, a more pronounced impact was observed, causing a 30.1% decrease in MSI and increases of 42.5% in H_2_O_2_ and 65.8% in MDA (**Table 2**). Main effects of genotypes demonstrated significant variability, with GS4 and Pegah exhibiting the highest MSI levels and the lowest H_2_O_2_ and MDA levels. In contrast, Payam and Sepideh displayed the lowest MSI levels and the highest H_2_O_2_ and MDA values (**Table 2**). Salinity and genotype interactions were significant, showing variations in hydrogen peroxide, MDA, and MSI levels. Under 60 mM NaCl, Payam had the highest percentage increase in hydrogen peroxide (33%), while GS4 had the lowest (4%). Sepideh had the highest increase in MDA (51%), while GS4 had the lowest (20%). The most substantial reduction in MSI was in Payam (20%), while Pegah and GS4 showed the most negligible reduction (9%). Under 120 mM NaCl, Sepideh had the highest percentage increase in hydrogen peroxide (57%), with GS4 having the lowest (20%). Payam showed the highest increase in MDA (91%), while Pegah had the lowest (40%) (**Figures 1B–D**). These findings underscore genotype-specific responses to salinity-induced stress, revealing significant differences in oxidative stress management and membrane stability.

### 3.3 Photosynthesis attributes

**Figure 2:**
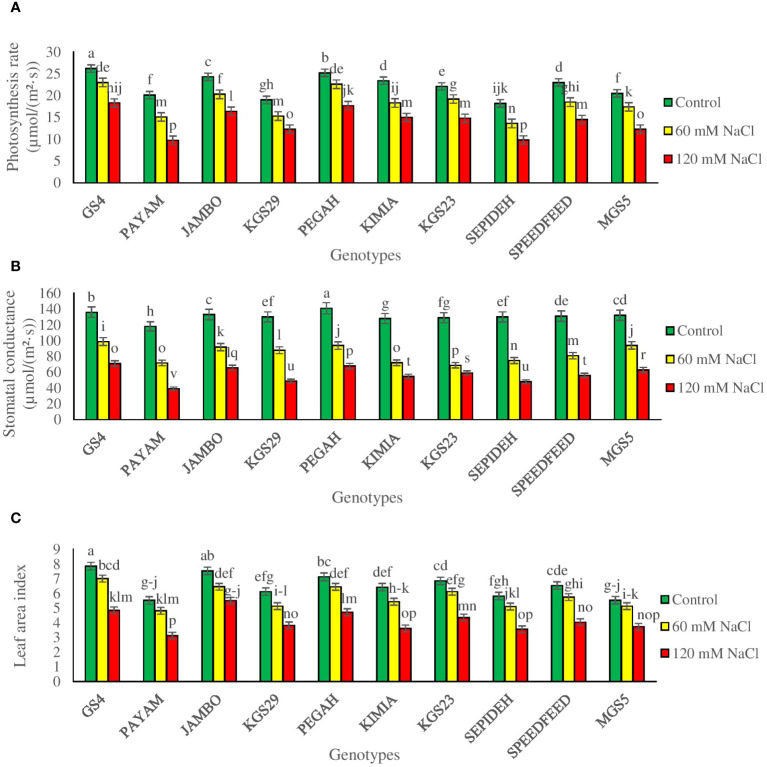
**(A)** photosynthesis rate (Pn), **(B)** stomatal conductance (Gs), and **(C)** leaf area index (LAI) of sorghum genotypes for interaction of genotypes × salinity (0, 60 and 120 mM NaCl) at fifty percent flowering stage. Different letters indicate significant differences by HSD at p<0.05.

The study underscores the substantial influence of salinity and genotypes on pivotal physiological parameters, including Pn, Gs, Ci, and LAI in sorghum plants. Notably, both main effects and their interaction were found to be statistically significant (**Supplementary Table 2**). In general, salinity exerted a discernible impact across all sorghum genotypes, leading to reductions in Pn, Gs, and LAI. Specifically, exposure to 60 mM NaCl resulted in a decrease of 17.2% in Pn, 36.6% in Gs, 27.1% in Ci, and 11.1% in LAI (**Table 2**). The deleterious effects intensified with 120 mM NaCl, causing a further reduction of 36.6% in Pn, 56.4% in Gs, 47.1% in Ci, and 22.0% in LAI (**Table 2**). Moreover, when scrutinizing the main effects of genotypes, GS4 and Pegah demonstrated the highest values in Pn, Gs, Ci, and LAI, underscoring their relative resilience to salinity stress. Conversely, Payam and Sepideh exhibited the lowest values among all sorghum genotypes, indicating heightened susceptibility (**Table 2**). However, the interaction effect between salinity and genotypes shows that the responses to salinity stress varied among sorghum genotypes (**Figures 2A–C**). At a salinity level of 60 mM NaCl, some genotypes exhibited a more modest decline in Pn (e.g., Payam: 25%) and Gs (e.g., GS4: 27%), indicating their relative tolerance to salinity. In contrast, others were more susceptible, with more significant reductions in these parameters (e.g., Kimia: 44% reduction in LAI). At a higher salinity level of 120 mM NaCl, the differences among genotypes became even more apparent, with some genotypes experiencing substantial reductions in Pn (e.g., Payam: 51%) and Gs (e.g., Payam: 67%), while others exhibited milder declines (e.g., GS4: 30% reduction in Pn).

### 3.4 Photosynthetic pigments content

**Figure 3:**
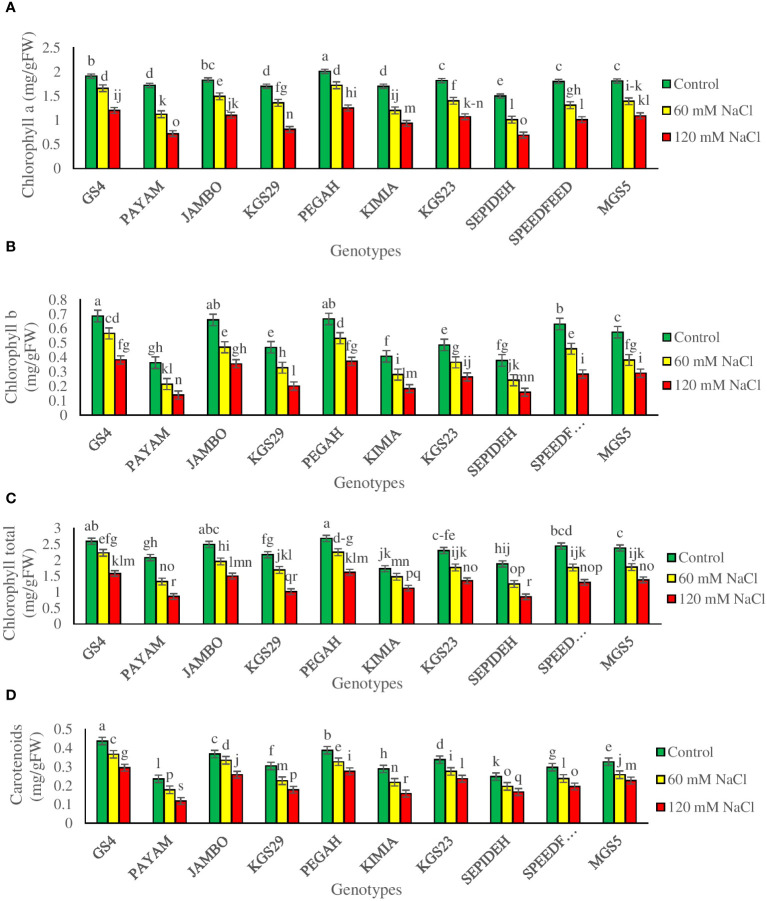
The contents of **(A)** chlorophyll a (Chl a), **(B)** chlorophyll b (Chl b), **(C)** total chlorophyll (Chl T) and **(D)** carotenoids (Car) of sorghum genotypes for interaction of genotypes × salinity (0, 60 and 120 mM NaCl) at fifty percent flowering stage. Different letters indicate significant differences by HSD at p<0.05.

The study revealed significant impacts of salinity and genotypes, with notable interactions, on photosynthetic pigment concentrations in sorghum plants (**Supplementary Table 2**). Salinity notably influenced chlorophyll a (Chl a), chlorophyll b (Chl b), total chlorophyll (Chl T), and carotenoids (Car) across sorghum genotypes. Exposure to 60 mM NaCl resulted in reductions of 23.6% in Chl a, 27.8% in Chl b, 22.9% in Chl T, and 18.9% in Car. At 120 mM NaCl, reductions intensified to 44.3% in Chl a, 50.2% in Chl b, 44.9% in Chl T, and 34.6% in Car (**Table 2**). Genotypic effects showed GS4 and Pegah with the highest pigment concentrations, while Payam and Sepideh had the lowest values. Noteworthy variations in pigment changes were observed among genotypes. At 60 mM NaCl, Payam displayed the highest chlorophyll reduction (35%), while GS4 had the lowest (13%). Chl b reductions varied, with Payam experiencing the most significant decrease (41%) and GS4 the least (17%). Chl T concentration variations were observed, with Payam undergoing the highest reduction (36%) and GS4 the lowest (13%). KGS29 had the most substantial Car reduction (26%), while Jumbo showed the least (9%) at this salinity level. At 120 mM NaCl, Payam showcased the highest chlorophyll reduction (58%), contrasting with GS4’s lowest reduction (37%). Chl b reductions varied, with Payam having the highest (62%) and Pegah the lowest (44%). Chl T concentration again saw Payam with the most significant reduction (59%) and Kimia with a relatively lower reduction (35%). Regarding Car, Payam displayed the highest percentage reduction (50%), while Pegah had a less pronounced reduction (29%) (**Figures 3A–D**). These findings underscore genotype-specific responses to varying salinity levels, emphasizing the intricate interplay between genetic traits and environmental stress.

### 3.5 Final yield

**Figure 4:**
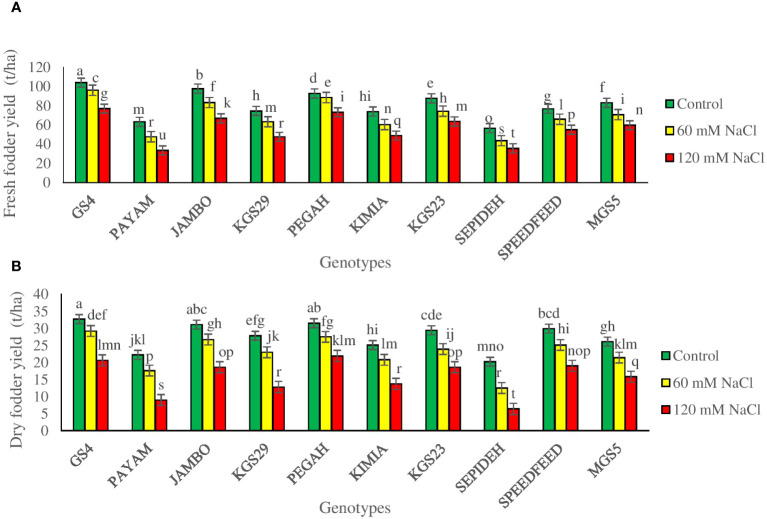
**(A)** Fresh fodder yield (FFY), and **(B)** dry fodder yield (DFY) of sorghum genotypes for the interaction of genotypes × salinity (0, 60, and 120 mM NaCl) at the fifty percent flowering stage. Different letters indicate significant differences by HSD at p<0.05.

To assess the impact of salt stress on sorghum genotypes, we measured fresh and dry fodder yields, crucial indicators of crop productivity. The study revealed significant main effects of salinity and genotypes, as well as notable interaction effects, on the final yield (**Supplementary Table 2**). Salinity significantly influenced both fresh and dry fodder yields in all sorghum genotypes. Exposure to 60 mM NaCl led to a 14.5% reduction in fresh yield and a 17.7% reduction in dry yield, while 120 mM NaCl caused more pronounced decreases of 30.8% in fresh yield and 43.4% in dry yield (**Table 2**). The main effect of genotypes showed that GS4 and Pegah had the highest values for both fresh and dry fodder yields, while Payam and Sepideh displayed the lowest values (**Table 2**). This emphasizes the inherent variability among sorghum genotypes in their ability to maintain final yield under salinity stress. The complex interaction between salinity and genotype significantly affected fresh and dry fodder yields. Across various salinity levels, all genotypes experienced consistent reductions in fresh and dry fodder yields. However, the extent of yield reduction varied notably among different sorghum genotypes when subjected to salinity stress. At 60 mM NaCl, the Payam genotype exhibited the most significant decreases in fresh and dry fodder yields, with 23% and 33% reductions, respectively. In contrast, the Pegah genotype showed milder reductions, with declines of 10% in fresh yield and 12% in dry yield. Upon increasing salinity to 120 mM NaCl, the Payam genotype sustained the highest reductions in both fresh (47%) and dry (58%) fodder yields. Conversely, the Pegah genotype displayed the least reduction, with declines of 21% in fresh yield and 30% in dry yield (**Figures 4A, B**). These variations in yield responses to salinity stress among sorghum genotypes highlight the intricate interplay between genetic factors and external stressors, providing valuable insights into sorghum’s adaptive capabilities in challenging environments.

### 3.6 Salinity tolerance

**Figure 5:**
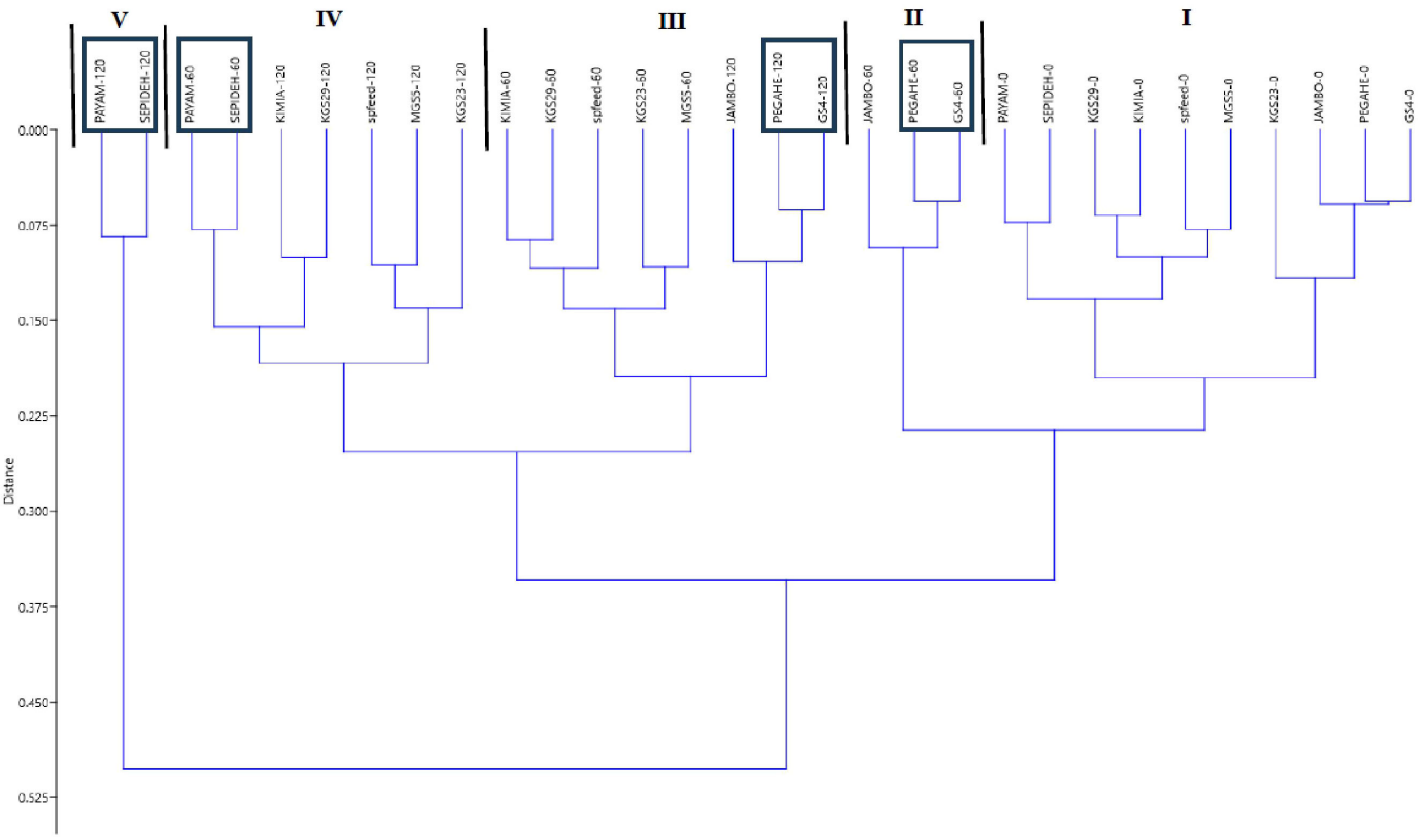
The outcomes of the hierarchical agglomerative cluster analysis, considering all traits across the three salt treatments. Clusters I–V are formed based on the similarity observed in all parameters. The samples exhibiting the highest sensitivity to salt stress within each cluster are highlighted within a frame. PEGAH-0 (genotype)–salinity level (mM NaCl).

In this study, we employed SSI and STI as reliable criteria to assess the salinity tolerance of sorghum genotypes. Dry matter yield was used to calculate these indices (**Table 3**). At 60 mM NaCl, the Sepideh genotype exhibited the highest SSI value (2.15), whereas the GS4 genotype showed the lowest SSI value (0.605). Conversely, the GS4 genotype displayed the highest STI value (1.25), while the Sepideh genotype had the lowest STI value (0.331). Moving to 120 mM NaCl, the Sepideh genotype demonstrated the highest SSI value (1.57), while the Pegah genotype showed the lowest (0.700). On the other hand, the Pegah genotype exhibited the highest STI value (0.905), and the Sepideh genotype had the lowest STI value (0.169). These findings indicate that the Pegah and GS4 genotypes displayed the highest tolerance to salinity, as evidenced by their lower SSI values and higher STI values. In contrast, the Sepideh and Payam genotypes were the most sensitive among the ten tested genotypes, with higher SSI and lower STI values. In addition, based on the classification tree analysis, the genotypes Payam and Sepideh exhibited the highest sensitivity to salt stress, while GS4 and Pegah demonstrated the highest tolerance (**Figure 5**). Under normal conditions (control), Payam and Sepideh clustered with the other genotypes. However, when exposed to 60 mM NaCl stress, Payam and Sepideh displayed distinct responses and formed a separate cluster (group IV). Notably, the samples grown under 60 mM NaCl were clustered with the genotypes subjected to 120 mM NaCl stress (group IV). Further, Payam and Sepideh under 120 mM NaCl stress constituted a separate cluster (group V), exhibiting the lowest growth parameters. In contrast, GS4 and Pegah clustered with the other genotypes under 60 mM stress and demonstrated better growth performance under 120 mM NaCl stress (cluster III, **Figure 5**).

**Table 3:** SSI and STI indices of sorghum cultivars and genotypes under the influence of different salinity levels.

Index	SSI		STI	
Salinity levels (mM NaCl)	60	120	60	120
Genotypes				
GS4	0.605	0.850	1.25	0.884
PAYAM	1.17	1.37	0.513	0.260
JAMBO	0.799	0.924	1.09	0.759
KGS29	0.975	1.24	0.839	0.467
PEGAH	0.717	0.700	1.14	0.905
KIMIA	0.968	1.04	0.685	0.451
KGS23	1.06	0.844	0.922	0.718
SEPIDEH	2.15	1.57	0.331	0.169
SPEEDFEED	0.907	1.06	0.985	0.746
MGS5	1.02	0.907	0.733	0.541

### 3.7 Defense mechanisms

#### 3.7.1 Ionic homeostasis

**Figure 6:**
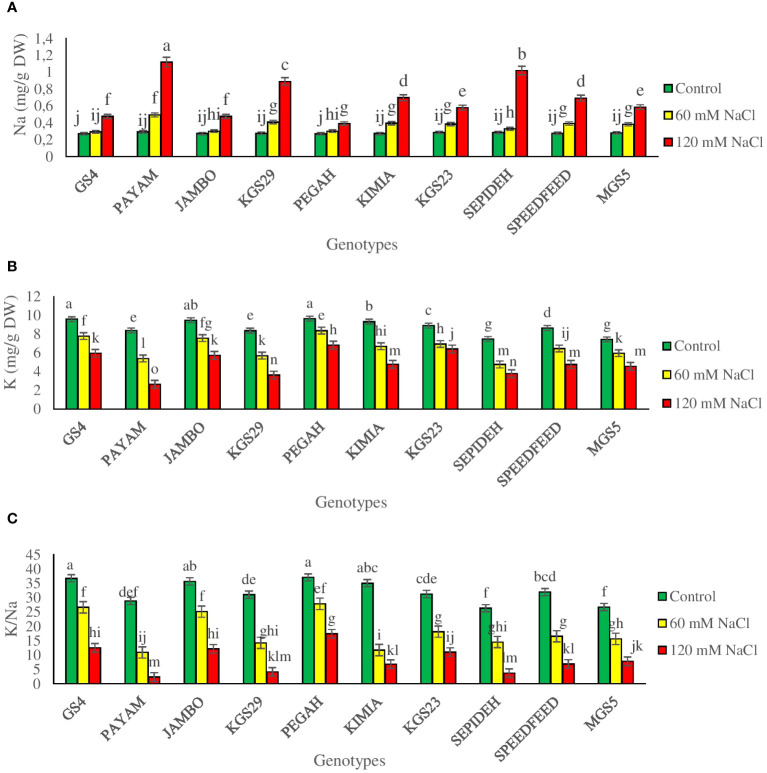
**(A)** sodium concentration (Na), **(B)** potassium concentration (K) and **(C)** potassium to sodium ratio (K/Na) of sorghum genotypes for interaction of genotypes × salinity (0, 60 and 120 mM NaCl) at fifty percent flowering stage. Different letters indicate significant differences by HSD at p<0.05.

The study emphasized significant main effects of salinity and genotypes, with notable interaction effects, on Na^+^ and K^+^ concentrations and the K^+^/Na^+^ ratio (**Supplementary Table 2**). Salinity consistently reduced K^+^ concentrations and the K^+^/Na^+^ ratio while increasing Na^+^ levels across sorghum genotypes. Exposure to 60 mM NaCl resulted in a 24.8% decrease in K^+^, a 43.4% reduction in the K^+^/Na^+^ ratio, and a 32% increase in Na+ (**Table 2**). At 120 mM NaCl, these effects intensified, causing a 30.8% decrease in K^+^, a 43.9% reduction in the K^+^/Na^+^ ratio, and a substantial 149% increase in Na^+^ (**Table 2**). Examining the main effect of genotypes, GS4 and Pegah had the highest K^+^ concentrations and K^+^/Na^+^ ratio while having the lowest Na^+^ levels. Conversely, genotypes Payam and Sepideh displayed the lowest K^+^ concentrations and K^+^/Na^+^ ratio, with the highest Na^+^ values. The interaction effect highlighted dynamic responses to salinity stress concerning Na^+^ and K^+^ concentrations and the K^+^/Na^+^ ratio. Specifically, the Payam genotype exhibited the most substantial surge in Na content at 60 mM NaCl (69%), while the GS4 genotype showed the least pronounced elevation (8%). At 120 mM NaCl, Payam and Pegah showcased the highest and lowest increments in Na^+^ content (283% and 45%, respectively), with similar trends observed in K^+^ reduction and the K^+^/Na^+^ ratio (**Figures 6A–C**). These findings underscore the genotype-specific nature of responses to salinity stress.

#### 3.7.2 Osmolytes

**Figure 7:**
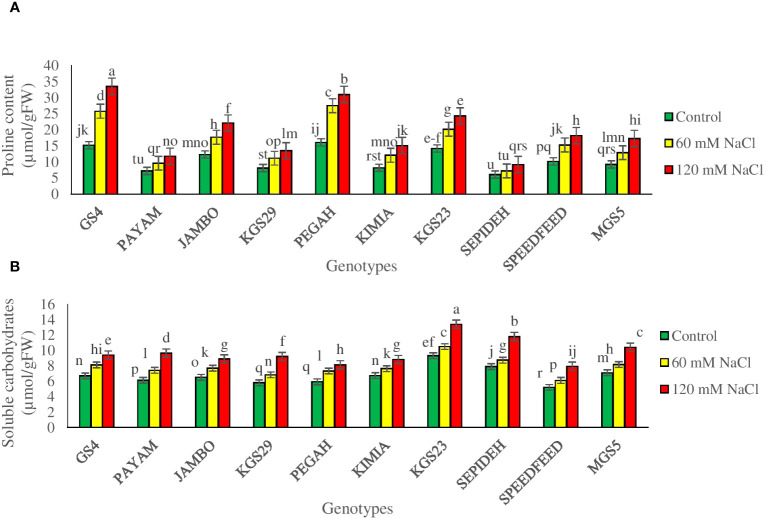
The contents of **(A)** proline (P) and **(B)** soluble carbohydrates (Carbo) of sorghum genotypes for the interaction of genotypes × salinity (0, 60, and 120 mM NaCl) at the fifty percent flowering stage. Different letters indicate significant differences by HSD at p<0.05.

The study revealed significant main effects of salinity and genotypes on P accumulation and Carbo levels. Salinity consistently increased P and Carbo concentrations across sorghum genotypes (**Supplementary Table 2**). Exposure to 60 mM NaCl resulted in a 50.0% increase in P and a 16.7% rise in Carbo, while 120 mM NaCl intensified these effects, causing an 84.9% increase in P and a 45.2% rise in Carbo (**Table 2**). Analyzing the main effect of genotypes showed that GS4 and Pegah had the highest P accumulation and Carbo levels, while Payam and Sepideh exhibited the lowest values for both P accumulation and Carbo levels (**Table 2**). The investigation revealed a significant interaction between salinity levels and genotypic responses, affecting P accumulation and Carbo levels (**Supplementary Table 2**). As salinity concentrations increased, a consistent trend emerged across all genotypes, marked by increased P and Carbo concentrations. However, the extent of this increase varied distinctly among genotypes. At 60 mM NaCl, Pegah and GS4 genotypes exhibited notable 71% and 70% increases in P concentration, respectively. In contrast, the Payam genotype showed a more modest 33% increment, and the Sepideh genotype had an 18% elevation in P levels. With 120 mM NaCl exposure, Pegah experienced a substantial 92% rise in P concentration, while GS4 recorded a marked increase of 120%. The Payam genotype demonstrated a 63% increment, and the Sepideh genotype exhibited a 51% elevation in P concentration (**Figure 7A**). Regarding Carbo, at 60 mM NaCl, Pegah and GS4 genotypes registered 24% and 21% increases, respectively. Under 120 mM NaCl, Pegah demonstrated a heightened increase of 37%, while GS4 showed a substantial 40% rise (**Figure 7B**). The Payam genotype displayed a 58% increment, and the Sepideh genotype revealed a 49% elevation in Carbo concentration. Notably, Pegah and GS4 genotypes exhibited robust osmoregulation mechanisms, while the Payam and Sepideh genotypes displayed comparatively more restrained responses.

#### 3.7.3 Antioxidant enzymes activities

**Figure 8:**
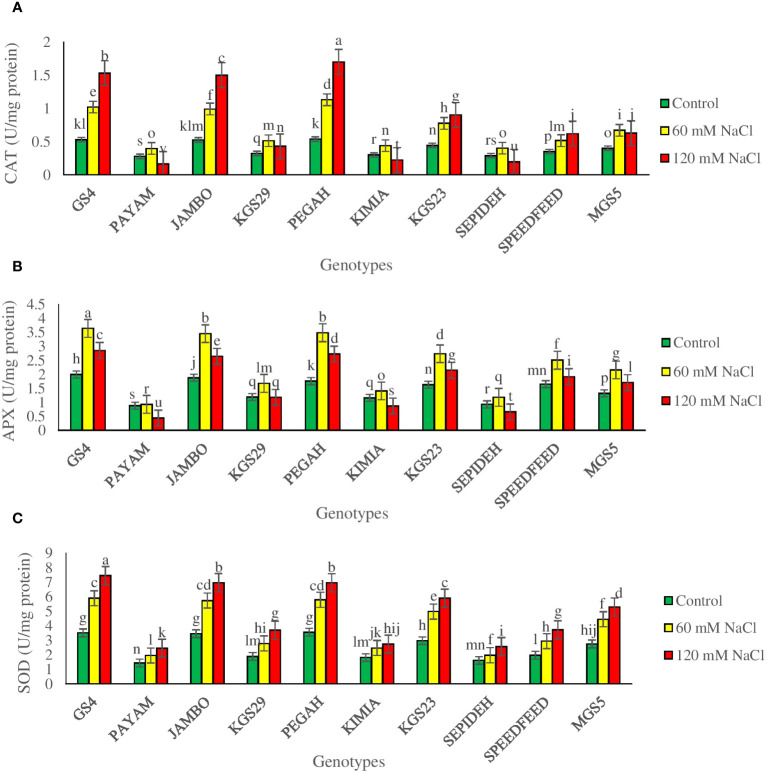
The specific activity of antioxidative enzymes **(A)** catalase (CAT), **(B)** ascorbate peroxidase (APX), and **(C)** super-oxide dismutase (SOD) of sorghum genotypes for the interaction of genotypes × salinity (0, 60, and 120 mM NaCl) at fifty percent flowering stage. Different letters indicate significant differences by HSD at p<0.05.

The investigation unveiled significant main effects of both salinity and genotypes on antioxidant enzyme activities (**Supplementary Table 2**). Salinity consistently led to increased catalase (CAT), superoxide dismutase (SOD), and ascorbate peroxidase (APX) activities across all sorghum genotypes. Exposure to 60 mM NaCl resulted in a 74.6% increase in CAT, a 56.2% rise in SOD, and a 61.1% elevation in APX (**Table 2**). A higher salinity level of 120 mM NaCl intensified these effects, causing a 95.4% increase in CAT, a 92.7% rise in SOD, and a more modest 18.7% increase in APX, which was lower than the lower salt level (**Table 2**). Analyzing the main effect of genotypes revealed that among all sorghum genotypes, GS4 and Pegah showcased the highest values for CAT, SOD, and APX activities. In contrast, genotypes Payam and Sepideh exhibited the lowest activities for these antioxidant enzymes. These findings highlight the inherent variability among sorghum genotypes in their ability to modulate antioxidant defense mechanisms in response to salinity stress. We also observed a significant interplay between salinity levels and genotypic responses, profoundly impacting the activities of antioxidant enzymes CAT, APX, and SOD (**Table 3**). Under moderate salinity conditions, all examined genotypes show heightened activities of these enzymes. However, as stress levels escalate, distinct response patterns surface across genotypes (**Figure 8**). CAT activity varies considerably among genotypes under varying salinity conditions. The Pegah genotype exhibits a marked surge in CAT activity (110%), in contrast to the Sepideh genotype, which shows a modest increment (39%). At the salinity level of 120 mM NaCl, CAT activity responses diverge. Notably, the Pegah genotype displays the most substantial increase (216%), while the Payam genotype demonstrates the most notable reduction (41%) (**Figure 8A**). APX activity also exhibits significant fluctuations among genotypes. The Pegah genotype showcases a substantial upsurge (98%), while the Payam genotype exhibits a modest increment (5%). At the same salinity level, APX activity varies among genotypes, with Pegah displaying the highest percentage increment (55%) and Payam showing the most considerable decline (50%) (**Figure 8B**). SOD activity uniformly increases across all genotypes at the salinity level of 120 mM, with notable variations in the magnitude of the increase. The GS4 genotype depicts the highest percentage rise (112%), while the Kimia genotype displays a relatively more modest increment (51%) (**Figure 8C**). These dynamics underscore the intricate genotypic responses to salinity stress and the potential implications of heightened antioxidant enzyme activities in augmenting salinity tolerance. Significantly, SOD consistently increases, demonstrating enhanced resilience and efficacy under severe stress conditions, while CAT and APX display heightened sensitivity to variations in stress intensity.

### 3.8 Principal components analysis

**Figure 9:**
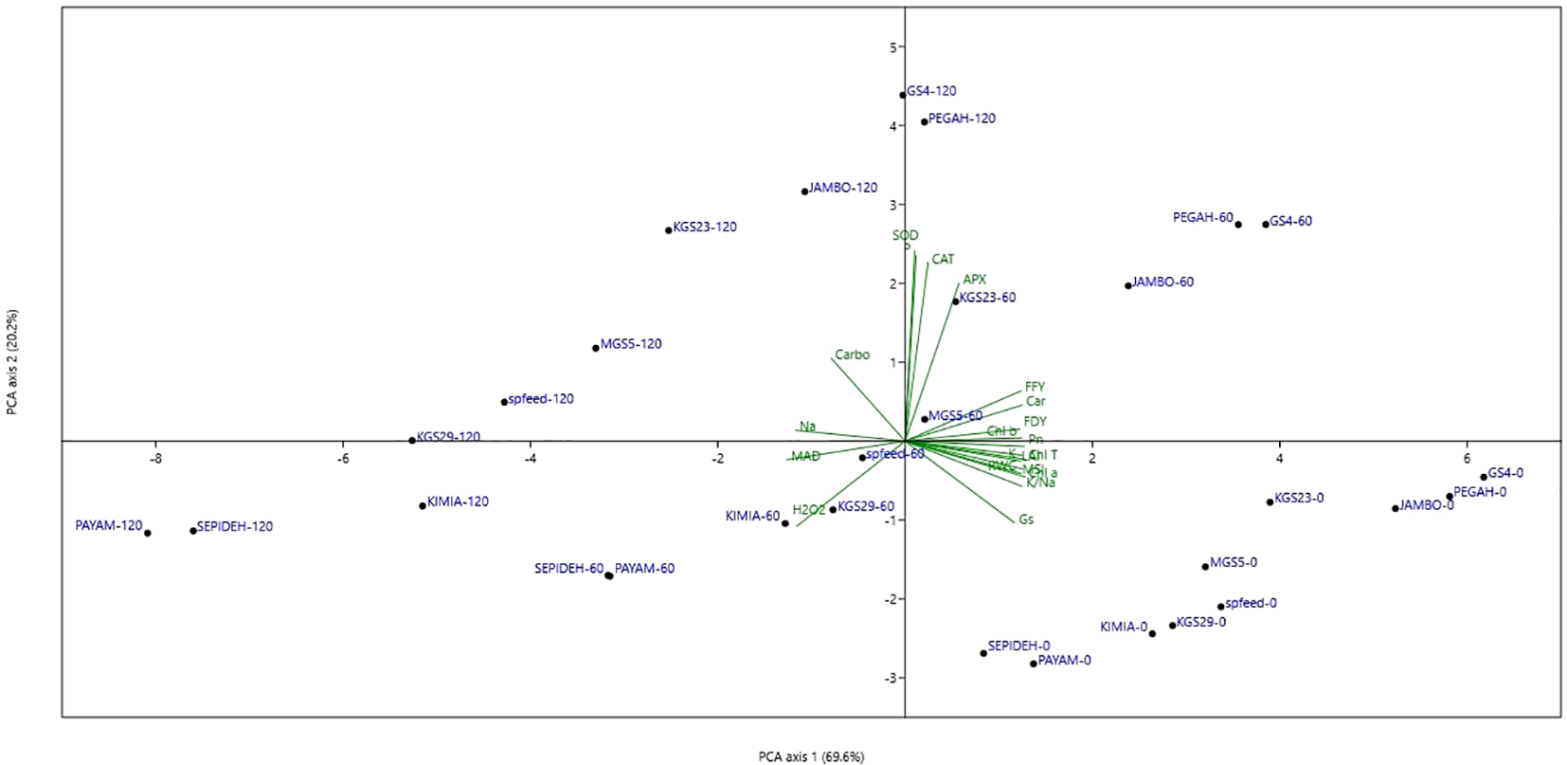
Principal components analysis diagram (PCA) of ten sorghum genotypes in three salinity treatments. RWC, relative leaf water content; Na, sodium content; K, potassium content; K/Na, K to Na ratios in the shoot; H_2_O_2_, hydrogen peroxide concentration; MDA, malondialdehyde concentration; MSI, membrane stability index; Pn, photosynthetic rate; Gs, stomatal conductance; Ci, intercellular CO_2_ concentration; LAI, leaf area index; Chl a, chlorophyll a; Chl b, chlorophyll b; Chl T, total chlorophyll; Car, carotenoids; P, proline; Carbo, soluble carbohydrates; CAT, catalase; APX, ascorbate peroxidise; SOD, superoxide dismutase; FFY, fresh fodder yield; DFY, day fodder yield; PEGAH-0–genotype -salinity level Mm NaCl.

Principal Components Analysis (PCA) was employed to enhance the graphical representation of distinct salt-genotype responses, as illustrated in **Figure 9**. The first principal component (PC1) distinctly accounted for 69.6% of the variance, delineating a salinity gradient across experimental conditions, ranging from right (0 mM NaCl treatments) to left (120 mM NaCl treatments). The second principal component (PC2), governing genotype responses, contributed to 20.2% of the total variance, revealing a hierarchical arrangement of genotypes from lower to upper positions on the diagram. Particularly noteworthy is the spatial distribution of sorghum genotypes under non-saline treatment, predominantly located on the lower right. At the same time, those exposed to 60 mM NaCl exhibited a dual distribution on the upper right and lower left. Genotypes treated with 120 mM NaCl primarily clustered on the left side of the scatter plot. The spatial arrangement on the PCA plot unveiled a correlation between treatments favoring higher growth on the right side and inferior performance under saline conditions on the left. Correlation analysis demonstrated that PC1 correlated positively with various physiological parameters, including K^+^, K^+^/Na^+^, RWC, MSI, Pn, LAI, Chl a, b, T, Car, FFY, FDY, APX, and Gs, while exhibiting negative correlations with H_2_O_2_, MDA, and Na^+^. In contrast, PC2 exhibited positive correlations with P, CAT, APX, and SOD. Furthermore, a positive association was observed between FFY and FDY and the activities of antioxidant enzymes, photosynthetic attributes, pigments, proline content, K^+^, and K^+^/Na^+^, while negative correlations were identified with Na^+^, MDA, and H_2_O_2_. These intricate relationships underscore the multifaceted interplay between salinity levels, sorghum genotypes, and their physiological responses. Notably, genotypes GS4 and Pegah demonstrated superior performance across all salt levels, while genotypes Payam and Sepideh exhibited suboptimal performance under all salinity conditions.

## 4 Discussion

This study delved into the responses of sorghum plants to salinity, focusing on genotype diversity and the mechanisms behind salt tolerance. In response to salinity, we consistently observed reductions in various crucial parameters such as RWC, K^+^ content, K^+^/Na^+^, MSI, Pn, Gs, Ci, LAI, Chl a, Chl b, Chl T and Car, fresh fodder yield, and dry fodder yield in all sorghum genotypes. Conversely, there were increases in Na^+^ content, H_2_O_2_ concentration, MDA concentration, P, Carbo, CAT, APX, and SOD across all sorghum genotypes. These findings corroborate existing research, highlighting how salinity impacts physiological, biochemical, and growth parameters, ultimately affecting overall yield (Tari et al., 2013; Ali et al., 2022; Rajabi Dehnavi et al., 2022). However, a rich tapestry of responses unfolded beyond this broad trend, exposing the genetic diversity among sorghum genotypes. This dynamic interplay between different salinity levels and genotypes responses underscores the intricate genetic adaptations that equip each genotype to confront adversity. These variations among genotypes are discernible through their unique physiological and biochemical reactions to salt stress, offering valuable insights into the mechanisms governing salt tolerance in sorghum.

Photosynthesis is a fundamental metabolic pathway crucial for regulating plant growth, which emerges as a primary target of salinity stress (Pessarakli, 2018; Pan et al., 2021). Our results show a strong positive correlation between Pn and final fresh and dry yields (r = 0.92 and 0.93, respectively) (**Supplementary Table 3**). Our findings consistently demonstrate a decline in Pn, Gs, and LAI under elevated salinity levels across various sorghum genotypes, emphasizing the common challenges of compromised gas exchange and hindered growth (Sharma et al., 2020; Rajabi Dehnavi et al., 2022). Detrimental effects of salinity on photosynthesis encompass multiple facets, including impacts on stomatal operations, gas exchange, pigments, chloroplast development, membrane structure, electron transport, enzyme activities and photosynthesis surface, ultimately impeding crop production (Ashraf and Harris, 2013; Pan et al., 2021; Amombo et al., 2022). However, our observations reveal that salt-tolerant genotypes (e.g., Pegah and GS4) effectively maintained their Pn and mitigated the negative effects of salt stress on growth. This is evidenced by the minimal reduction in Pn and yields in these genotypes. The preservation of photosynthetic efficiency can be traced through the impact of salinity on stomatal and non-stomatal factors. We noted genotype-specific Gs, LAI and pigment responses, underscoring the adaptive diversity within sorghum genotypes. Salt-tolerant genotypes adeptly retained Gs, LAI and pigments, while salt-sensitive genotypes exhibited greater susceptibility, resulting in a more pronounced decline in photosynthetic activity. This divergence probably results from genotype-specific variations in their ability to handle stress-induced disorders, especially the ability of these genotypes to inhibit oxidative stress levels, managing water-related stress and maintenance ionic homeostasis. To prove theses hypothesis, we delved into the intricate dynamics governing cellular membrane responses to oxidative stress induced by salt exposure. In this regard, we used a comprehensive approach that includes the assessment of hydrogen peroxide levels, MSI, and MDA concentrations, which gained deeper insights into the complex interplay involving genetic diversity, salt stress, and cellular reactions. Our findings highlight significant correlations between elevated hydrogen peroxide and MDA levels and the imposition of salt stress, aligning with established knowledge that links increased levels of ROS to subsequent cellular damage. Concurrently, the reduction in MSI values, indicating decreased membrane stability, underscores the susceptibility of cellular membranes to oxidative stress. These patterns are consistent with observations in other plant species, emphasizing the universality of these stress-responsive mechanisms (Youssef et al., 2021; Anwar-ul-Haq et al., 2023). However, our investigation unveils genotype-specific variations, providing insights into the underlying defense mechanisms. The substantial increase in hydrogen peroxide and MDA concentrations in the salt-sensitive genotypes (e.g., Payam and Sepideh), coupled with decreased MSI, signifies its heightened vulnerability to oxidative stress and membrane damage. In contrast, the observed lower levels of hydrogen peroxide and MDA, along with enhanced membrane stability, in the salt-tolerance genotypes (e.g., Pegah and GS4) underscore their superior ability to withstand oxidative stress. These findings emphasize the significance of MDA and MSI as reliable indicators of salt tolerance. These results confirm our hypothesis about the ability of salt-tolerant genotypes to control oxidative stress. However, to understand the mechanism of this ability, we investigated the antioxidant defense system more closely. Our exploration revealed intriguing trends in the activities of CAT, APX, and SOD, presenting a multifaceted view of antioxidant defense responses to salinity stress. The strong positive correlations between sorghum yield and antioxidant enzymes activities highlight the importance of an efficient enzymatic antioxidant defense system in salt-tolerant genotypes (**Supplementary Table 3**). In addition, we observed that while SOD consistently showed higher activity under stress, CAT and APX exhibited different reactions to varying stress levels. This contrast suggests inherent genetic variability in the efficiency of the enzymatic antioxidant defense system among different sorghum genotypes. SOD enhanced stability and efficacy under severe stress contrast with CAT and APX greater sensitivity to stress intensity variations. These observations underscore the dynamic nature of plant defense mechanisms, which adapt elegantly to the nuances of salinity stress.

In addition, it seems that the orchestration of osmoregulation, along with the modulation of antioxidant enzyme activity, plays a central role in facilitating sorghum plants adaption and resilience to salinity stress. Osmotic regulation plays a crucial role in maintaining water balance and structural integrity. Proline and Carbo, critical players in the osmotic stress response, are vital components of plant adaptive strategies (Alagoz et al., 2023). The positive correlations between P concentration and both fresh (r=0.71) and dry (r=0.70) fodder yield indicated the positive impact of proline accumulation on photosynthesis and cell stability (**Supplementary Table 3**). Proline, acting as a compatible solute, reinforces cell membrane and protein stability while serving as an effective antioxidant and regulator of cellular processes (Shafi et al., 2019). Simultaneously, soluble carbohydrates sustain turgor pressure and assist water absorption in saline conditions (Singh et al., 2015). It has been documented that under saline conditions, there is an augmentation in the activity of proline synthesis enzymes, including pyrroline carboxylic acid and glutamyl kinase (Zulfiqar and Ashraf, 2023). Consequently, there is a noticeable elevation in proline content within cells. Furthermore, the observed surge in soluble carbohydrate content is likely a result of disruptions in their synthesis, transport, and utilization pathways. These disruptions inevitably lead to the breakdown of complex sugars (Varshney et al., 2023).

Variations in RWC among different sorghum genotypes subjected to varying salinity levels provide valuable insights into their strategies for managing water-related stress. The consistent reduction in RWC under higher salinity levels underscores the significant impact of osmotic stress, leading to decreased water availability and subsequent adjustments in cellular turgor. These distinct RWC responses across genotypes highlight the complex interplay between genetic traits and environmental conditions, collectively shaping a plant ability to retain water. This observation aligns with previous studies recognizing RWC as an informative marker of stress-induced water deficits (Irigoyen et al., 1992; Zhang et al., 2022). Our findings reveal that salt-tolerant genotypes Pegah and GS4 maintain higher RWC values, indicating their better ability to preserve water under salinity-induced stress. Conversely, salt-sensitive genotypes Payam and Sepideh exhibit lower RWC values, suggesting their reduced capacity to retain water in high salinity conditions. This difference in RWC responses highlights the ability of salt-tolerant genotypes to counteract salt-induced water deficits, possibly due to osmoregulation ability and improved cell wall integrity (Irigoyen et al., 1992; Zhang et al., 2022).

In addition, our findings strongly reinforce the critical significance of K^+^/Na^+^ ratio as a key indicator of salt tolerance (Basu et al., 2021; Balasubramaniam et al., 2023). A higher K^+^/Na^+^ ratio serves as a hallmark of effective ion management, reducing the influx of harmful ions while promoting the retention of essential nutrients. This phenomenon aligns with salt-tolerant plants, which naturally possess an enhanced ability to maintain an elevated K^+^/Na^+^ ratio, limiting Na^+^ absorption and enhancing K^+^ assimilation (Saqib et al., 2011; Amombo et al., 2022; Balasubramaniam et al., 2023). The distinct responses exhibited by salt-tolerant sorghum genotypes, particularly exemplified by Pegah and GS4, in maintaining an optimal K^+^/Na^+^ ratio underscore their adaptability to saline conditions. These genotypes likely employ a range of strategies, including restricted Na^+^ uptake, increased K^+^ acquisition, and coordinated regulation of ion transport, to enhance their performance under salt-induced stress. This finding emphasizes the well-established importance of ion homeostasis in unraveling the intricate mechanisms of salt tolerance (Amtmann and Sanders, 1998; Nieves-Cordones et al., 2016; Mansour et al., 2021; Balasubramaniam et al., 2023).

Our investigation delves into the genotype-specific modulation of salinity responses, as discerned through the comprehensive analysis of the Principal Components Analysis (PCA) diagram. Under control conditions, the sorghum genotypes exhibited uniform behavior, indicating a baseline consistency in their responses. However, the introduction of salt stress unveiled distinctive and significant shifts in their responses. Notably, the salt-tolerant genotypes, prominently represented by GS4 and Pegah, showcased a superior and adaptive performance under salt stress, particularly in the challenging conditions of high salinity (120 mM NaCl). Remarkably, the performance of these salt-tolerant genotypes rivaled that of other genotypes subjected to a lower level of salinity (60 mM NaCl), emphasizing their robust and versatile response mechanisms. The observed high positive correlation between the salt-tolerant genotypes and key physiological parameters, including antioxidant enzymes and osmolytes, serves as compelling evidence supporting our conclusions on the intricate mechanisms underpinning the salt tolerance of these sorghum genotypes. This correlation highlights the orchestrated interplay of various biochemical and physiological pathways that contribute to the enhanced adaptability of genotypes to salt-induced stress. Conversely, the salt-sensitive genotypes, Payam and Sepideh, exhibited the lowest performance across both salt levels. The strong negative correlations identified with Na^+^, MDA, and H_2_O_2_ underscore the limited efficiency of the defense mechanisms in these genotypes against salt stress. This vulnerability suggests a compromised ability to regulate ion homeostasis and manage oxidative stress, crucial aspects in mitigating the impact of salinity. These findings provide valuable insights into the complex relationships between salinity levels, specific sorghum genotypes, and the intricate physiological responses that govern their adaptation to salt stress. Such nuanced understanding is crucial for informing targeted strategies in crop breeding and management practices to enhance salt stress resilience in sorghum cultivation.

In summary, the highlighted defense mechanisms enabled salt-tolerant genotypes to effectively counteract ionic toxicity, maintain a favorable K^+^/Na^+^ ratio, sustain optimal photosynthesis, improve stomatal function, preserve membrane integrity, and ensure sufficient levels of photosynthetic pigments. This resulted in the maintenance of photosynthetic capacity, improved growth conditions, and stable performance under salt stress. In contrast, sensitive genotypes struggled to deploy these defenses efficiently, rendering them highly susceptible to salinity stress. Additionally, the study delved into the nuanced specificity of sorghum genotypes, revealing diverse reactions across physiological and biochemical parameters. Salinity’s significant impact spanned Relative Water Content (RWC), photosynthetic pigments, physiological parameters (Pn, Gs, Ci, LAI), ion concentrations (Na^+^, K^+^), final yield (FFY, DFY), and antioxidant enzyme activities (CAT, SOD, APX). In terms of RWC, GS4 and Pegah excelled in water retention, contrasting with the lower resilience of Payam and Sepideh to salinity-induced water stress. Analysis of photosynthetic pigments underscored GS4 and Pegah’s ability to maintain higher concentrations, highlighting their vitality compared to the vulnerability of Payam and Sepideh. Physiological parameters emphasized GS4 and Pegah’s enhanced photosynthetic efficiency and overall resilience, while Payam and Sepideh faced challenges in mitigating salinity’s impact. Ion concentrations further showcased GS4 and Pegah’s adaptability with higher K^+^ concentrations and K^+^/Na^+^ ratios, along with lower Na^+^ levels, differentiating them from the more susceptible Payam and Sepideh. Final yield evaluation confirmed GS4 and Pegah’s resilience with higher FFY and DFY values, indicating suitability for saline environments. Analysis of antioxidant enzyme activities solidified GS4 and Pegah’s robust defense mechanisms against salinity stress, contrasting with the weaker response of Payam and Sepideh.

## 5 Conclusion

This study explored salinity stress responses in sorghum, focusing on genotype variations and salt tolerance mechanisms. Salt-tolerant sorghum genotypes demonstrated effective osmotic regulation, strong antioxidant enzyme activity, and a maintained K^+^/Na^+^ ratio, ensuring stable photosynthesis, stomatal function, and membrane integrity. These mechanisms contributed to performance maintenance and reduced yield loss. The study identified key indicators such as K^+^/Na^+^ ratio, MDA, MSI, SOD, and proline for distinguishing between tolerant and sensitive genotypes, offering valuable insights for sorghum breeding. The findings support the potential cultivation of salt-tolerant sorghum in saline areas, enhancing sustainable sorghum production for food security in challenging environments. Pegah and GS4 emerged as promising candidates for salt-affected environments, warranting further testing across different locations and years for reliable yield assessments.

## Data availability statement

The raw data supporting the conclusions of this article will be made available by the authors, without undue reservation.

## Author contributions

ARD: Conceptualization, Data curation, Formal analysis, Investigation, Methodology, Software, Validation, Visualization, Writing – original draft. MZ: Conceptualization, Methodology, Supervision, Validation, Visualization, Writing – review & editing. AP: Formal analysis, Validation, Visualization, Writing – review & editing.
