# Supplementary material for: Understanding salinity stress responses in sorghum: exploring genotype variability and salt tolerance mechanisms
Source: Front Plant Sci. 2024 Jan 9;14:1296286. doi: 10.3389/fpls.2023.1296286 (PMC10806974; doi:10.3389/fpls.2023.1296286)
Supplement: Supplementary file 1 [file Table_1.docx]

**Supplementary Figure 1. (A)** Changes in minimum daily temperature (Tmin), maximum daily temperature (Tmax) during the sorghum cultivation period in 2021 based on the statistics of Najaf Abad weather station, **(B)** Daily relative humidity (RH) and daily potential evapotranspiration (ETo) during the growth period of sorghum plant in 2016 based on the statistics of Najaf Abad weather station.
