# Supplementary material for: Understanding salinity stress responses in sorghum: exploring genotype variability and salt tolerance mechanisms
Source: Front Plant Sci. 2024 Jan 9;14:1296286. doi: 10.3389/fpls.2023.1296286 (PMC10806974; doi:10.3389/fpls.2023.1296286)
Supplement: Supplementary file 2 [file Table_2.docx]

**Supplementary Table 1.** List of abbreviations

| **Abbreviation** | **Full description** |
| --- | --- |
| ANOVA | Analysis of variance |
| APX | Ascorbate peroxidase |
| Car | carotenoids |
| Carbo | soluble carbohydrates |
| CAT | catalase |
| Chl a | chlorophyll a |
| Chl b | chlorophyll b |
| Chl T | total chlorophyll |
| Ci | intercellular CO2 concentration |
| DFY | Dry forage yield |
| EC | electrical conductivity |
| FFY | Fresh forage yield |
| Gs | stomatal conductance |
| HSD | Tukey's honest significance test |
| K^+^ | potassium content |
| K^+^/Na^+^ | K to Na ratios |
| LAI | leaf area index |
| MDA | malondialdehyde |
| MSI | membrane stability index |
| Na^+^ | sodium content |
| P | Proline |
| PCA | Principal Components Analysis |
| Pn | photosynthetic rate |
| RAW | The readily available water |
| ROS | reactive oxygen species |
| RWC | The relative water content |
| SOD | superoxide dismutase |
| SSI | Stress sensitivity index |
| STI | Salinity tolerance index |
| TAW | Total available water |
| TBA | Thiobarbituric Acid |
| TCA | Trichloroacetic Acid |
| TVW | The volume of water required for irrigation |

**Supplementary Table 2.** Analysis of variances (mean squares) for different parameters of 10 sorghum Genotypes in Three levels salinity.

| Trait | Sources of Variations | | | | |
| --- | --- | --- | --- | --- | --- |
|  | Salinity | Main Error | Genotypes | Salinity*Genotypes | Error |
| Df | 2 | 6 | 9 | 18 | 54 |
| RWC | 5462* | 0.855 | 245** | 79.8** | 1.22 |
| Na | 1.43** | 0.002 | 0.089** | 0.052** | 0.0003 |
| K | 110* | 0.65 | 9.51** | 0.831** | 0.024 |
| K/Na | 4202** | 100 | 192** | 18.9* | 5.61 |
| H2O2 | 133** | 0.047 | 122** | 3.82** | 0.041 |
| MDA | 485** | 2.27 | 92.9** | 7.89** | 1.22 |
| MSI | 4404** | 0.206 | 341** | 25.3** | 0.150 |
| Pn | 489** | 0.816 | 75.4** | 1.19** | 0.280 |
| Gs | 42479** | 1.96 | 614** | 80.5** | 1.36 |
| Ci | 7770** | 41.1 | 362** | 61.9 n.s | 44.1 |
| LAI | 15.2** | 0.111 | 4.46** | 0.201* | 0.101 |
| Chl a | 4.68** | 0.020 | 0.293** | 0.015** | 0.002 |
| Chl b | 0.540** | 0.002 | 0.111** | 0.002** | 0.0005 |
| Chl T | 7.81** | 0.033 | 0.804** | 0.031* | 0.015 |
| Car | 0.095** | 0.0001 | 0.033** | 0.0003** | 0.0003 |
| P | 604** | 2.44 | 335** | 18.0** | 0.786 |
| Carbo | 71.18* | 0.011 | 15.85** | 0.59** | 0.011 |
| CAT | 1.20** | 0.0001 | 0.940** | 0.188** | 0.0009 |
| APX | 6.08** | 0.0002 | 5.25** | 0.334** | 0.0005 |
| SOD | 40.6** | 0.023 | 19.9** | 1.06** | 0.031 |
| FFY | 4687** | 0.327 | 2155** | 18.3** | 0.296 |
| DFY | 1093** | 2.60 | 197** | 3.50* | 1.11 |

RWC= relative leaf water content, Na= sodium content, K= potassium content, K/Na= K to Na ratios in shoot, H2O2= hydrogen peroxide concentration; MDA= malondialdehyde concentration, MSI= membrane stability index, Pn= photosynthetic rate; Gs= stomatal conductance; Ci= intercellular CO2 concentration; LAI= leaf area index; Chl a = chlorophyll a; Chl b = chlorophyll b; Chl T=total chlorophyll; Car = carotenoids; P= proline; Carbo= soluble carbohydrates; CAT = catalase; APX = ascorbate peroxidise; SOD = superoxide dismutase; FFY = fresh fodder yield; DFY= day fodder yield; df = degrees of freedom; ns = non-significant; Error = within group variance;**= P ≤ 0.01 * = P ≤ 0.05.

**Supplementary Table 3.** Correlation coefficients between treats for different parameters of sorghum genotypes under saline conditions (for each parameter n = 3).

| No. | parameters | 1 | 2 | 3 | 4 | 5 | 6 | 7 | 8 | 9 | 10 | 11 | 12 | 13 | 14 | 15 | 16 | 17 | 18 | 19 | 20 |
| --- | --- | --- | --- | --- | --- | --- | --- | --- | --- | --- | --- | --- | --- | --- | --- | --- | --- | --- | --- | --- | --- |
| 1 | CAT | 1 |  |  |  |  |  |  |  |  |  |  |  |  |  |  |  |  |  |  |  |
| 2 | APX | 0.78^**^ | 1 |  |  |  |  |  |  |  |  |  |  |  |  |  |  |  |  |  |  |
| 3 | SOD | 0.92^**^ | 0.79^**^ | 1 |  |  |  |  |  |  |  |  |  |  |  |  |  |  |  |  |  |
| 4 | Na | -0.51^n.s^ | -0.71^*^ | -0.37^n.s^ | 1 |  |  |  |  |  |  |  |  |  |  |  |  |  |  |  |  |
| 5 | K | 0.62^*^ | 0.80^**^ | 0.56^n.s^ | -0.81^**^ | 1 |  |  |  |  |  |  |  |  |  |  |  |  |  |  |  |
| 6 | RWC | 0.59^n.s^ | 0.76^**^ | 0.46^n.s^ | -0.94^**^ | 0.81^**^ | 1 |  |  |  |  |  |  |  |  |  |  |  |  |  |  |
| 7 | Carbo | -0.05^n.s^ | -0.23^n.s^ | 0.18^n.s^ | 0.47^n.s^ | -0.20^n.s^ | -0.41^n.s^ | 1 |  |  |  |  |  |  |  |  |  |  |  |  |  |
| 8 | Proline | 0.88^**^ | 0.75^**^ | 0.90^**^ | -0.34^n.s^ | 0.56^n.s^ | 0.39^n.s^ | 0.09^n.s^ | 1 |  |  |  |  |  |  |  |  |  |  |  |  |
| 9 | H2O2 | -0.84^**^ | -0.94^**^ | -0.82^**^ | 0.74^*^ | -0.83^**^ | -0.80^**^ | 0.25^n.s^ | -0.79^**^ | 1 |  |  |  |  |  |  |  |  |  |  |  |
| 10 | MDA | -0.70^*^ | -0.88^**^ | -0.67^*^ | 0.80^*^ | -0.87^**^ | -0.86^**^ | 0.26^n.s^ | -0.66^*^ | 0.94^**^ | 1 |  |  |  |  |  |  |  |  |  |  |
| 11 | MSI | 0.49^n.s^ | 0.81^**^ | 0.43^n.s^ | -0.90^**^ | 0.77^**^ | 0.91^**^ | -0.45^n.s^ | 0.37^n.s^ | -0.80^**^ | -0.88^**^ | 1 |  |  |  |  |  |  |  |  |  |
| 12 | Pi | 0.58^n.s^ | 0.87^**^ | 0.51^n.s^ | -0.83^**^ | 0.89^**^ | 0.81^**^ | -0.47^n.s^ | 0.57^n.s^ | -0.85^**^ | -0.87^**^ | 0.86^**^ | 1 |  |  |  |  |  |  |  |  |
| 13 | Gs | 0.36^n.s^ | 0.70^*^ | 0.25^n.s^ | -0.87^**^ | 0.77^**^ | 0.88^**^ | -0.56^*^ | 0.21^n.s^ | -0.69^*^ | -0.77^**^ | 0.90^**^ | 0.81^**^ | 1 |  |  |  |  |  |  |  |
| 14 | LAI | 0.46^n.s^ | 0.81^**^ | 0.36^n.s^ | -0.85^**^ | 0.84^**^ | 0.87^**^ | -0.45^n.s^ | 0.33^n.s^ | -0.74^*^ | -0.79^**^ | 0.87^**^ | 0.91^**^ | 0.86^**^ | 1 |  |  |  |  |  |  |
| 15 | Chl a | 0.51^n.s^ | 0.85^**^ | 0.45^n.s^ | -0.86^**^ | 0.88^**^ | 0.87^**^ | -0.48^n.s^ | 0.46^n.s^ | -0.84^**^ | -0.89^**^ | 0.90^**^ | 0.93^**^ | 0.92^**^ | 0.90^**^ | 1 |  |  |  |  |  |
| 16 | Chl b | 0.63^*^ | 0.94^**^ | 0.60^*^ | -0.77^**^ | 0.78^**^ | 0.80^**^ | 0.44^n.s^ | 0.59^*^ | -0.89^**^ | -0.86^**^ | 0.89^**^ | 0.91^**^ | 0.84^**^ | 0.87^**^ | 0.92^**^ | 1 |  |  |  |  |
| 17 | Chl T | 0.56^n.s^ | 0.90^**^ | 0.51^n.s^ | -0.85^**^ | 0.87^**^ | 0.86^**^ | -0.47^n.s^ | 0.51^n.s^ | -0.89^**^ | -0.89^**^ | 0.91^**^ | 0.94^**^ | 0.91^**^ | 0.91^**^ | 0.99^**^ | 0.96^**^ | 1 |  |  |  |
| 18 | Car | 0.74^*^ | 0.96^**^ | 0.74^*^ | -0.77^**^ | 0.87^**^ | 0.81^**^ | -0.22^n.s^ | 0.69^*^ | -0.92^**^ | -0.91^**^ | 0.85^**^ | 0.88^**^ | 0.78^**^ | 0.85^**^ | 0.88^**^ | 0.94^**^ | 0.92** | 1 |  |  |
| 19 | FFY | 0.72^*^ | 0.96^**^ | 0.73^*^ | -0.77^**^ | 0.88^**^ | 0.80^**^ | -0.28^n.s^ | 0.71^*^ | -0.95^**^ | -0.94^**^ | 0.85^**^ | 0.92^**^ | 0.78^**^ | 0.83^**^ | 0.92^**^ | 0.95^**^ | 0.95^**^ | 0.98^**^ | 1 |  |
| 20 | DFY | 0.55^n.s^ | 0.88^*^ | 0.49^n.s^ | -0.83^**^ | 0.83^**^ | 0.83^**^ | -0.56^*^ | 0.70^*^ | -0.86^**^ | -0.89^**^ | 0.89^**^ | 0.93^**^ | 0.83^**^ | 0.87^**^ | 0.95^**^ | 0.92^**^ | 0.96^**^ | 0.86^**^ | 0.92^**^ | 1 |
